# Long Non-Coding RNAs as Biomarkers and Therapeutic Targets in Sepsis

**DOI:** 10.3389/fimmu.2021.722004

**Published:** 2021-09-22

**Authors:** Chuqiao Wang, Guorui Liang, Jieni Shen, Haifan Kong, Donghong Wu, Jinxiang Huang, Xuefeng Li

**Affiliations:** ^1^The Sixth Affiliated Hospital of Guangzhou Medical University, Qingyuan People’s Hospital, State Key Laboratory of Respiratory Disease, Sino-French Hoffmann Institute, School of Basic Medical Sciences, Guangzhou Medical University, Guangzhou, China; ^2^Nanshan School, Guangzhou Medical University, Guangzhou, China; ^3^Shenzhen Luohu People’s Hospital, The Third Affiliated Hospital of Shenzhen University, Shenzhen, China

**Keywords:** sepsis, long non-coding RNAs, biomarkers, therapeutic targets, organ injury

## Abstract

Sepsis, an infection-induced systemic inflammatory disorder, is often accompanied by multiple organ dysfunction syndromes with high incidence and mortality rates, and those who survive are often left with long-term sequelae, bringing great burden to social economy. Therefore, novel approaches to solve this puzzle are urgently needed. Previous studies revealed that long non-coding RNAs (lncRNAs) have exerted significant influences on the process of sepsis. The aim of this review is to summarize our understanding of lncRNAs as potential sepsis-related diagnostic markers and therapeutic targets, and provide new insights into the diagnosis and treatment for sepsis. In this study, we also introduced the current diagnostic markers of sepsis and discussed their limitations, while review the research advances in lncRNAs as promising biomarkers for diagnosis and prognosis of sepsis. Furthermore, the roles of lncRNAs in sepsis-induced organ dysfunction were illustrated in terms of different organ systems. Nevertheless, further studies should be carried out to elucidate underlying molecular mechanisms and pathological process of sepsis.

## Introduction

Sepsis is characterized as an unbalanced immune response to infection, potentially developing into multiple organ dysfunction syndrome and threatening human lives (1). Millions of patients, particularly older individuals, cancer patients and immunocompromised patients, suffer from sepsis every year (2–4). According to the World Health Organization (WHO), sepsis and sepsis-induced shock are recognized as serious public health problems (5). Apart from this, sepsis becomes the leading cause of mortality for patients in the intensive care unit (6). Emerging data from clinical studies have indicated that hospitalized patients with septic shock were associated with mortality rates of up to 40% (7). Sepsis is often associated with poor prognosis, and even patients who are discharged from the hospital will be accompanied with various long-term sequelae, including mental illness, cognitive impairment, and cardiovascular disease, which has a tremendous impact on global health and social economy (8).

Traditionally, sepsis was mainly treated with fluid resuscitation, antibiotic therapy, lung protective ventilation, and blood purification due to the lack of specific medications (9–11). However, on account of the complexity of the host immune response and the pathophysiological mechanisms involved during sepsis, the therapeutic outcomes of these treatments were not as effective as expected. Although 258 sepsis-related biomarkers have been identified during the past ten years, none of these biomarkers showed sufficient sensitivity or specificity to be pervasively applied in clinical practice (12, 13). The development for current treatment of sepsis is mainly focused on regulating inflammatory imbalance, immune dysfunction, and coagulopathy (12). Despite the fact that clinical trials for finding promising novel strategies for sepsis have been underway, lack of preclinical research has limited the knowledge translating from bench to bedside (14, 15). Hence, there is an urgent need to find new diagnostic markers and identify therapeutic targets so as to accurately stratify the stages of sepsis, facilitate early diagnosis, and develop personalized treatment.

Non-coding RNAs (ncRNAs), including circular RNAs, microRNAs (miRNAs) and long non-coding RNAs (lncRNAs) have been identified as regulatory RNAs that present the ability of interacting with key mediators in many biological processes (16), as well as the potential of becoming novel medical markers or therapeutic targets for sepsis. Without the ability of protein-coding, lncRNAs refer to ncRNAs more than 200 nt in length (17). Compared with messenger RNAs, lncRNAs have lower expression levels and poorer sequence conservation, with specificity related to cell types, organs and disease process (17–19). Relevant studies have reported that lncRNAs were involved in a wide range of biological activities, such as genomic imprinting, chromosome modification and silencing, transcriptional activation and repression (through regulating gene expression in epigenetics, transcription, and translation) (18–20). The molecular functions of lncRNAs are summarized into four archetypes, including guides, scaffolds, decoys, and signals (21). In addition to this, recent research reported that the specific expression of lncRNAs could regulate cellular development, metabolism, and differentiation, thereby involving in a variety of human disease (22). Moreover, other studies also showed that lncRNAs differentially expressed according to different process of sepsis, in the lipopolysaccharide (LPS)-treated renal tubular epithelial cells, LPS-treated cardiomyocytes, LPS-treated monocytes, as well as LPS-treated endothelial cells (23–26). Nonetheless, the investigations about the involvement of lncRNAs in sepsis are limited. In this paper, the biogenesis of lncRNA was briefly introduced and the limitations of current biomarkers for sepsis were discussed. Furthermore, we summarized the roles of lncRNAs in sepsis and introduced new promising lncRNAs that might act as diagnostic and prognostic markers for sepsis. Finally, in order to gain a more comprehensive understanding of the pathogenesis of sepsis, we also summarized the roles of lncRNAs in sepsis-induced organ dysfunction in terms of different organ systems.

## The Roles of lncRNAs in Sepsis

Pathological conditions of patients with sepsis will change over time, initially characterizing as inflammatory response, accompanied by the secretion of numerous proinflammatory cytokines (e. g., tumor necrosis factor [TNF]-α, interleukin [IL]-6, IL-8, and IL-1β) (27), followed by the secretion of a large number of anti-inflammatory cytokines (e. g., IL-10), then transitioning to the phase of immunosuppression (28). During the immunosuppressive phase, proliferative capacity of lymphocytes decreased, making the body more susceptible to pathogenic infection (29). Hence, clear diagnosis and effective treatment during the early phase of sepsis will significantly improve clinical outcomes.

In recent years, although many literatures have pointed out that the aberrant expression of lncRNAs were involved in occurrence of many diseases, the links between lncRNAs and sepsis have still been not completely understood. One study showed that lncRNA metastasis-associated lung adenocarcinoma transcript (MALAT) 1 promoted TNF-α expression in LPS-treated cardiomyocytes (30). Apart from that, a positive correlation was observed between the expression of lncRNA nuclear-enriched abundant transcript (NEAT) and TNF-α in LPS-induced macrophages (31). In the peripheral blood cells of patients with sepsis, the upregulated lncRNA colorectal neoplasia differentially expressed (CRNDE) expression levels were associated with the poor prognosis of sepsis (32). According to research by Huang et al. (33), the expression of lncRNA downregulated in liver cancer stem cells (DILC) was downregulated in peripheral blood and LPS-treated THP-1 cells, resulting in upregulated IL-6 expression. These studies directly or indirectly suggested that lncRNAs had significant relationship with sepsis.

Recently, some research has showed that lncRNAs modulated sepsis by regulating different signaling pathways, such as Toll-like receptor (TLR) signaling pathway (34), which activated nuclear factor κB (NF-κB), thereby triggered inflammatory response in sepsis. A Previous study has suggested that lncRNA hox transcript antisense RNA (HOTAIR) activated the NF-κB signaling pathway to promote TNF-α expression in a murine septic model (35). Likewise, CRNDE and NEAT1 promoted the progression of sepsis-induced injury by regulating the NF-κB signaling (34, 36).

**Figure 1:**
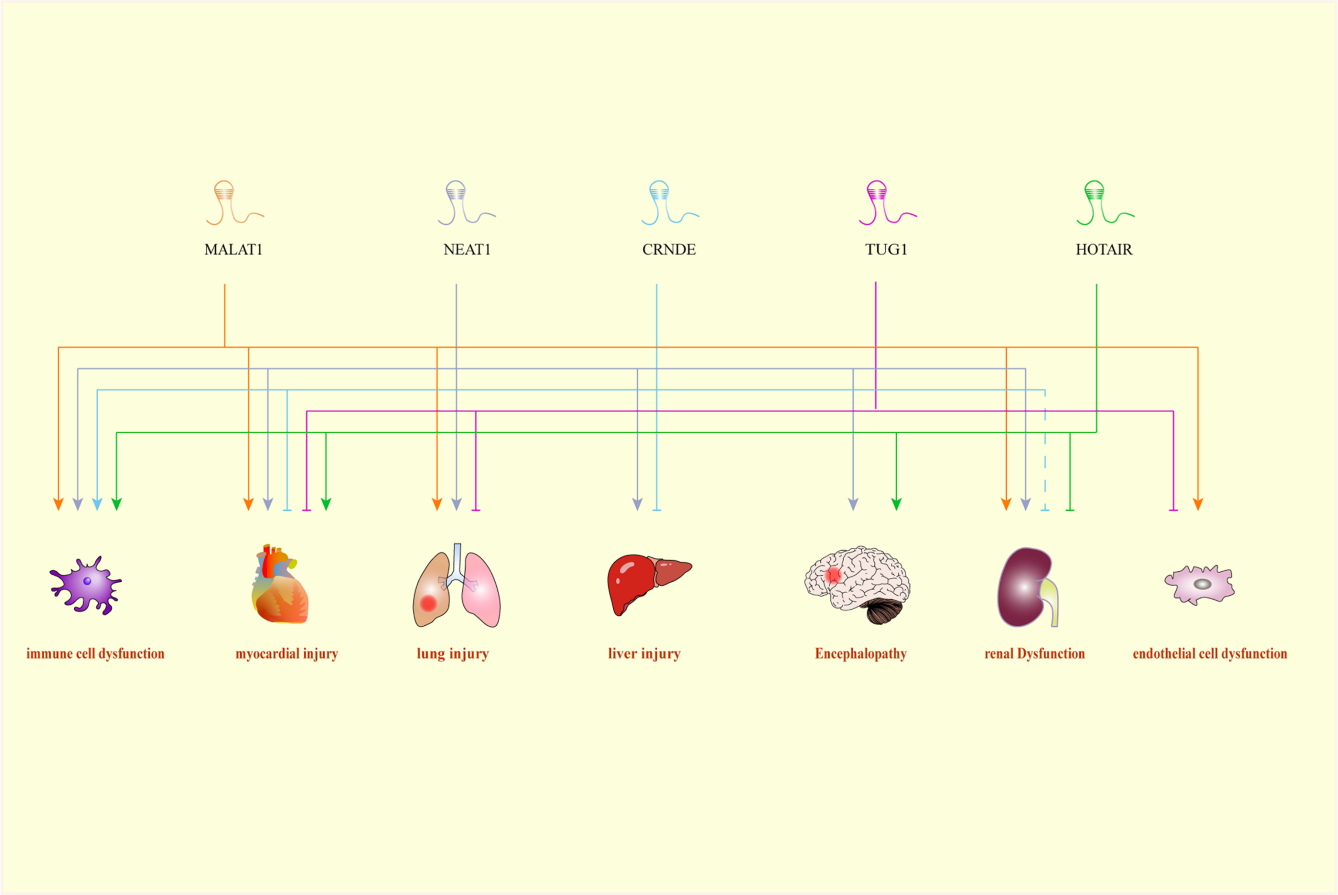
A Comprehensive association between LncRNAs and their target organs. CRNDE, colorectal neoplasia differentially expressed; HOTAIR, HOX transcript antisense RNA; MALAT1, metastasis-associated lung adenocarcinoma transcript 1; NEAT1, nuclear-enriched abundant transcript 1; TUG1, Taurine up-regulated gene 1.

Moreover, the interaction between miRNAs and lncRNAs is becoming one of the hot points currently. It has been reported that HOTAIR inhibited the apoptosis of kidney cells in a rat model of sepsis through downregulating of miR-34a/B-cell lymphoma 2 (Bcl-2) signaling pathway (37). Another study conducted by Chen et al. also convinced the involvement of HOTAIR in the etiology of sepsis (38). HOTAIR promoted sepsis progression through sponging miR-211 and inducing the expression of IL-6 receptor (IL-6R) (38). Taurine up-regulated gene (TUG) 1 could alleviate sepsis-triggered inflammation and cellular apoptosis *via* targeting miR-34b-5p and GRB2 associated binding protein (GAB) 1 (39). Previous study reported that MALAT1 bound to miR-23a to upregulate the expression of mast cell-expressed membrane protein (MCEMP) 1, hence promoting the inflammatory response in sepsis (40), indicating that lncRNAs could affect the development of sepsis through diverse signaling pathways. Negatively regulated by MALAT1, hsa-miR-346 played a vital role in the progression of sepsis by inhibiting the expression of SMAD3 (41). Working as the sponge of miR-125, NEAT1 might upregulate MCEMP1 to promote inflammatory factor activities as well as apoptosis, which might serve as a novel biomarker for treatment of septic patients (42, 43). Another *in vitro* study demonstrated that NEAT1 was observed to regulate miR-495-3p/signal transducers and activators of transcription axis and miR-211/phosphoinositide-3-kinase/protein kinase B axis in inflammatory process of sepsis (44). Even though the investigations for the roles of lncRNAs in septic shock are lacking, Wu et al. identified lncRNA THAP9-AS1 and TSPOAP1-AS1 that might be of practical values for pediatric septic shock diagnosis (45). What is more interesting is that some lncRNAs like MALAT1, NEAT1, CRNDE, TUG1, and HOTAIR could exert therapeutic or diagnostic influence across different organ systems (**Figure 1**). MALAT1 was reported to cause sepsis-mediated organic injuries by promoting apoptosis and enhancing immune response both *in vivo* and *in vitro*. Zhuang et al. found that MALAT1 induced by IL-6 could upregulate TNF-α expression through activating serum amyloid antigen 3 in cardiomyocytes, which could reduce myocardial contractility and cardiac function during sepsis (30). Besides, Yu et al. found that MALAT1 can induce apoptosis and hyperpermeability of cardiac microvascular endothelial cells (CMVECs), which exerting negative effects on the normal condition of adjacent cardiomyocytes and coronary microvessels (46). Additionally, lung is regarded as another critical and susceptible target organ during sepsis. Liang et al. revealed that MALAT1 increased TNF-α and IL-6 levels through the activation of the MyD88/NF-κB pathway by sponging miR-149, causing damage to alveolar epithelial cells (47). Nevertheless, the study by Lin et al. further demonstrated that MALAT1 could protect the lung from sepsis-induced injury *via* inhibiting NF-κB signaling (48). Furthermore, the expression of MALAT1 was elevated during sepsis, and thus inhibited cell proliferation and apoptosis in LPS-induced acute kidney injury (49).

Similar to MALAT1, NEAT1 can promote the progression of sepsis through promoting cell apoptosis, enhancing immune response, and decreasing cellular activity. Wang et al. revealed that knockdown of NEAT1 reduced the expression of inflammatory factors through inhibition of the TLR2/NF-κB pathway, thereby reduced cardiac tissue damage and improved cardiac function in sepsis-induced mice model (50). Subsequently, Wei et al. further demonstrated that NEAT1 could exacerbate cardiomyocyte injury *via* sponging miR-144-3p (51). Moreover, NEAT1 was reported to enhance the immune response through activation of the high-mobility group box 1 (HMGB1)/receptors for advanced glycation end products (RAGE) and NF-κB pathway, which exacerbated lung epithelial cell injury in sepsis mice (52). Subsequent studies in sepsis patients further demonstrated that the direct interaction of NEAT1 with the target miR-125a increased apoptosis of lung epithelial cells, which ultimately led to poor prognosis of ARDS (43). Chen et al. found that overexpression of NEAT1 could inhibit miR-204 and activate the NF-κB pathway, aggravating sepsis-induced mesangial cell injury (53). Moreover, Zhang et al. found that high expression of NEAT1 competitively bound to lethal-7 (Let-7) and targeted TLR4, consequently resulting in hepatic injury (36). In addition, Liu et al. confirmed that upregulated NEAT1 increased BAX expression and decreased Bcl-2 expression, resulting in decreased neuronal cell activity and brain injury in cecal ligation and puncture (CLP)-induced sepsis model (54).

While MALAT1 and NEAT1 promoted sepsis-induced inflammation in organ injury, TUG1 exhibited protective effects by inhibiting apoptosis, promoting cell proliferation, and downregulating immune response in sepsis. TUG1 was found to sponge miR-27a-3p, and enhance slit guidance ligand 2 (SLIT2) expression to inhibit apoptosis and autophagy of vascular endothelial cells (55). This finding is consistent with that of Wang et al. who observed the overexpression of TUG1 could decrease TNF-α expression level and reduce sepsis-induced apoptosis of cardiomyocytes *via* ing miR-27a, which had a protective effect against sepsis-induced myocardial injury (56). Consistent with the present results, Qiu et al. reported that TUG1 could attenuate sepsis-related acute lung injury by regulating miR-34b-5p/GAB1 axis, inhibiting immune response as well as apoptosispongs (39).

Interestingly, some lncRNAs can promote sepsis-induced injury in certain organs, while exerting protective influences on other organ systems. CRNDE was reported to be protective of myocardial and liver injury during sepsis (57, 58). The overexpression of CRNDE was reported to accelerate LPS-induced inflammatory response in kidney by activating TLR4/NF-κB signaling pathway (59). These results reflected the study of Sun et al. (1992) (60) who also found that inhibiting CRNDE could alleviate sepsis-induced kidney injury by blocking the activation of TLR3/NF-κB signaling. However, the study by Wang et al. demonstrated that overexpression of CRNDE could downregulate the expression of miR-181a-5p, thereby increased the expression of peroxisome proliferator activated receptor α (PPARα), and finally protected kidney from more severe injury through promoting cell proliferation and preventing cell apoptosis (61). It is worth mentioning that Chen et al. and Wu et al. discovered that HOTAIR was upregulated in LPS-induced septic mice, and promoted monocyte apoptosis *via* sponging miR-211 and increasing TNF-α production through p65/NF-κB pathway (35, 38). This finding is contrary to the study which has suggested that HOTAIR was downregulated in rats with CLP-induced sepsis and overexpression of HOTAIR could inhibit the apoptosis of kidney tissues, thereby relieved acute kidney injury (35). The overview with respect to the implications of lncRNAs as biomarkers in sepsis-induced organic dysfunction were showed in **Table 1**.

**Table 1:** LncRNAs as potential therapeutic targets for sepsis-induced organic dysfunction.

LncRNA	Target	Downstream pathway	Organ injury	Effects to sepsis	Reference
**BAFF**		NF-κB/MLCK/MLC	Gastrointestinal tract	Promote	Quan et al. (62)
**CASC2**	miR-144-3p	AQP1	Lung	Alleviate	Li et al. (63)
	miR-545-3p	PPARα	Kidney	Alleviate	Hu et al. (64)
	miR-155	NF-κB	Kidney	Alleviate	Wang et al. (65)
**CRNDE**	miR-29a	SIRT1	Heart	Alleviate	Zhu et al. (57)
	miR-126-5p	BCL2L2	Liver	Alleviate	Li et al. (58)
	miR-181-5p	PPARα	Kidney	Alleviate	Wang et al. (32)
		TLR3/NF-κB	Kidney	Promote	Sun et al. (60)
	miR-181-5p	TLR4	Immune cells	Promote	Wang et al. (32)
**Gas5**		Ccl1	Gastrointestinal tract	Promote	Ito et al. (66)
**H19**	miR-874	AQP1	Heart	Alleviate	Fang et al. (67)
**HOTAIR**		p65/NF-κB	Heart	Promote	Wu et al. (35)
	miR-34a	Bcl-2	Kidney	Alleviate	Jiang et al. (37)
	miR-211		Immune cells	Promote	Chen et al. (38)
**HOXA‐AS2**	miR-106b-5p	Wnt/β-catenin /NF-κB	Kidney	Alleviate	Wu et al. (68)
**HULC**	miR-128-3p	RAC-1	Endothelial cells	Promote	Yang et al. (69)
**KCNQ1OT1**	miR-192-5p	XIAP	Heart	Alleviate	Sun et al. (70)
**Lethe**		LC3-II	Brain	Alleviate	Mai et al. (71)
**LINC00472**	miR-373-3p	TRIM8	Liver	Promote	Li et al. (72)
**Lnc-5657**		Spns2	Lung	Promote	Liu et al. (73)
**MALAT1**		SAA3	Heart	Promote	Zhuang (74)
	miR-149	MyD88/NF-κB	Lung	Promote	Liang et al. (47)
		P38 MAPK /p56 NF-κB	Lung	Promote	Lin et al. (48)
	miR-370-3p	HMGB1	Kidney	Promote	Xu et al. (49)
		EZH2	Endothelial cells	Promote	Yu et al. (46)
	hsa-miR-346	SMAD3	Immune cells	Promote	Yang et al. (41)
	miR-23-3p		Immune cells	Promote	Ma et al. (40)
**MEG3**		NF-κB	Immune cells	Alleviate	Pan and He (75)
**MIAT**	miR−330−5p	TRAF6/NF−κB	Heart	Promote	Xing et al. (76)
**NEAT1**	miR-144-3p	NF-κB	Heart	Promote	Wei et al. (51)
		TLR2/NF−κB	Heart	Promote	Wang et al. (50)
	miR-125a		Lung	Promote	Yang et al. (43)
		HMGB1/RAGE	Lung	Promote	Zhou et al. (52)
		NF-κB	Brain	Promote	Liu et al. (54)
		Let-7a/TLR4	Liver	Promote	Zhang and Niu (36)
	miR-204	NF-κB	Kidney	Promote	Chen et al. (53)
	miR-370-3p	TSP-1	Immune cells	Promote	Xu et al. (31)
	miR-211	PI3K/AKT	Immune cells	Promote	Xia et al. (44)
	miR-495-3	STAT3	Immune cells	Promote	Xia et al. (44)
	miR-125a-5p	TRAF6/TAK1	Immune cells	Promote	Wang et al. (77)
	miR-125	MCEMP1	Immune cells	Promote	Chen et al. (42)
**PVT1**		Bcl-2	Heart	Alleviate	Zhang et al. (23)
		p38 MAPK	Immune cells	Promote	Zheng et al. (78)
**RMRP**	miR-1-5p	hsp70	Heart	Alleviate	Han et al. (79)
	miR-206	DDX5	Kidney	Promote	Zhang et al. (80)
**SNHG1**	miR-181a-5p	XIAP	Heart	Alleviate	Luo et al. (81)
**SNHG14**	miR-495-3p	HIPK1	Kidney	Promote	Yang et al. (82)
**SOX2OT**		SOX2	Heart	Promote	Chen et al. (83)
		SOX2	Brain	Promote	Yin et al. (84)
**TapSAKI**	miR-22	PTEN/TLR4/p65	Kidney	Promote	Shen et al. (85)
	miR-205	IRF3	Kidney	Promote	Han et al. (86)
**TCONS_00016233**	miR-22-3p	AIFM1	Kidney	Promote	Zhang et al. (87)
**THRIL**	miR-424	ROCK2	Lung	Promote	Chen et al. (88)
**TUG1**	miR-27a		Heart	Alleviate	Wang et al. (56)
	miR-34b-5p	GAB1	Lung	Alleviate	Qiu et al. (39)
	miR-145-5p		Lung	Alleviate	Lv et al. (89)
	miR-27a-3p	SLIT2	Endothelial cells	Alleviate	Dong et al. (55)
**Wfdc21**		STAT3/TLR4	Immune cells	Promote	Xie et al. (90)
**XIST**	miR-16-5p		Lung	Alleviate	Song et al. (91)
	miR-15a-5p	CUL3	Kidney	Promote	Xu et al. (92)

AIFM1, apoptosis-inducing factor mitochondrial -associated 1; AKT, Protein Kinase B; AQP1, Aquaporin 1; BAFF, B cell-activating factor; Bcl-2, B-cell lymphoma 2; BCL2L2, BCL2-like 2; CASC2, cancer susceptibility candidate 2; Ccl1, chemokine (C-C motif) ligand 1; CRNDE, colorectal neoplasia diﬀerentially expressed; CUL3, cullin 3; CYTOR, cytoskeleton regulator RNA; DDX5, DEAD-box helicase 5; DLX6-AS1, DLX6 antisense RNA 1; EZH2, Enhancer of Zeste Homolog 2 Protein; FOXA1, Hepatocyte Nuclear Factor 3-alpha; GAB1, GRB2 associated binding protein 1; Gas5, growth arrest-specific 5; HIPK1, homeodomain-interacting protein kinase 1; HMGB1, high-mobility group box 1; HOTAIR, HOX transcript antisense RNA; HOXA-AS2, HOXA cluster antisense RNA 2; hsp70, heat-shock proteins 70; HULC, highly upregulated in liver cancer; IRF3, interferon regulatory factor 3; KCNQ1OT1, KCNQ1 overlapping transcript 1; LC3-II, microtubule-associated protein 1 light chain 3-II; MALAT1, metastasis-associated lung adenocarcinoma transcript 1; MAPK, mitogen-activated protein kinase; MCEMP1, mast cell-expressed membrane protein 1; MEG3, maternally expressed 3; MIAT, myocardial infarction associated transcript; mTOR, mechanistic target of rapamycin; MyD88, myeloid differentiation factor 88; NEAT1, nuclear-enriched abundant transcript 1; NF-κB, nuclear factor κB; NLRP3, NLR family pyrin domain containing 3; PI3K, phosphoinositide-3-kinase; PPARα, Peroxisome proliferator-activated receptor-α; PTEN, phosphatase and tensin homolog protein; RAC-1, Rac family small GTPase 1; PVT1, plasmacytoma variant translocation 1; RAGE, receptors for advanced glycation end products; RMRP, RNA component of mitochondrial RNA processing endoribonuclease; ROCK2, Rho-associated coiled-coil containing protein kinase 2; SAA3, serum amyloid antigen 3; SIKIAT1, sepsis-induced kidney injury associated transcript 1; SIRT1, sirtuin 1; SNRHG1, small nucleolar RNA host gene 1; SLIT2, slit guidance ligand 2; SMAD3, small mothers against decapentaplegic homolog 3; SNHG, small nucleolar RNA host gene; SOX2, SRY-box transcription factor 2; SOX2OT, SOX2 overlapping transcript; Spns2, spinster homologue 2; STAT3, signal transducer and activator of transcription 3; TAK1, Transforming growth factor-activated kinase 1; TapSAKI, transcript predicting survival in AKI; THRIL, tumor necrosis factor and HNRNPL related immunoregulatory long non-coding RNA; TLR, Toll-like receptor; TRAF6, TNF Receptor-Associated Factor 6; TSP-1, thrombospondin-1; TRAF6, TNF Receptor-Associated Factor 6; TUG1, Taurine up-regulated gene 1; UCA1, urothelial carcinoma-associated 1; XIAP, X-linked inhibitor of apoptosis protein; XIST, X inactive-specific transcript.

## Current Diagnostic Markers of Sepsis and Limitations

Biomarkers generally refer to the objective measurement and evaluation of a certain characteristic response in normal physiological or pathological process, through which we can know the current biological process of the body. Although bacterial culture is the golden standard for differential diagnosis of sepsis, this method is time-consuming with a high false negative rate. Thus to find novel diagnostic markers during sepsis is still meaningful and needed.

C-reactive protein (CRP) is an acute inflammatory factor and is the most import biomarker of sepsis. With moderate degree of sensitivity, such as soluble TREM-1 (93), CRP not only increased in sepsis, but also showed an upward trend in other inflammatory and infectious diseases with poor specificity. Apart from that, procalcitonin (PCT) can be produced from cells and tissues during infection. In the past two decades, despite lacking definitive evidence to support the use of PCT as a biomarker of sepsis, established evidence supports its power to assist in managing sepsis patients. The use of antibiotics can be guided by measuring the content of PCT in patients with sepsis (94), however it has no significant effect on the diagnosis and treatment of sepsis (95), and PCT-guided antibiotic therapy reduces antibiotic exposure of upper respiratory tract infection patients. Furthermore, based on the concentration of PCT, we cannot confirm whether the patient has sepsis. IL-6 is a pleiotropic cytokine that is known to induce release of CRP in response to inflammation or infection (96–98). IL-6 usually increases earlier than that of CRP and PCT, making it a potential biomarker for early detection of sepsis (99). However, the concentration of IL-6 also increases in patients with other non-infectious diseases (100). CD64 is significantly increased after activation of the neutrophils in response to infection within a few hours (101). Thus, neutrophil CD64 is a useful marker for early diagnosis of sepsis. However, the measurement using flow cytometry makes the application of CD64 challenging. Interestingly, Subtype of CD14, presepsin, is an emerging and reliable biomarker of sepsis. Although there is insufficient evidence currently supporting the overall diagnostic accuracy compared with PCT or CRP, presepsin may still have advantages for early screening of sepsis, which is a good sign for development for new biomarkers (93). The diagnostic limitations of these sepsis markers force us to explore new diagnostic markers, especially those can distinguish between different organs dysfunction.

## LncRNAs in Diagnosis and Prognosis of Sepsis

ncRNAs are detected in various organs or tissues, indicating it has the potential to be sepsis-related organ damage biomarkers. At present, most clinical samples suitable for the detection of ncRNAs are patient blood, including serum, plasma and blood cells (102). LncRNAs in the blood can be quantified quickly using quantitative polymerase chain reaction (qPCR) without spending too much time as bacterial culture, and thus avoid delaying of the diagnosis. This presents the advantages of lncRNAs as diagnostic biomarkers for sepsis. Different lncRNAs vary in expression in different sepsis processes. For example, previous experimental results of endothelial cell transcriptome analysis of sepsis revealed that AL132709.5 lncRNA was significantly upregulated, while CTC-45916.1 lncRNA, 45916.1 lncRNA, and lncRNA XLOC_007697 showed a trend of downregulation (26). Moreover, lncRNA EGO and lncRNA HOTAIR myeloid-specific 1 arrived their peaks at 3 hours, while lncRNA IL7R arrived its peak at 24 hours in sepsis model (25), which suggested that lncRNAs could be ideal biological indicators for diagnosing and distinguishing different stages of sepsis in the future. In vitro, by using LPS stimulated peripheral blood mononuclear cells (PBMCs), we found that the expression of NEAT1 increased rapidly and reached its peak at 2 hours post LPS treatment (99), while PCT needed 12-48 hours to reach its peak, indicating that NEAT1 was an early response factor during infection (100). This suggested that NEAT1 could be used as a potential and effective marker for rapid diagnosis of sepsis. Huang et al. found that the level of NEAT1 in patients who died of sepsis was higher than that in patients who survived, with an AUC of 0.641, indicating that NEAT1 could predict the poor prognosis of the disease (103). In the meanwhile, the expression of NEAT1 was found to be positively correlated with Acute Physiology and Chronic Health Evaluation (APACHE) II score (103). Compared with patients who died of sepsis, survivors also showed higher expression of lncRNA ENST00000504301.1, and lower expression of ENST00000452391.1 (104). Similarly, one study found that the expression of CRNDE was positively correlated with sequential organ failure assessment (SOFA), APACHEII score, and the expression of CRP and PCT (61).

In addition to the aforementioned lncRNAs, other lncRNAs may also work as efficient diagnosis and prognosis biomarkers for sepsis. LncRNA MALAT1 was correlated with good prediction of sepsis and risk of 28-day death with an AUC of 0.823 and 0.755, respectively (105). In addition, the expression of MALAT1 was positively associated with APACHE II score (P < 0.001) and SOFA score (P < 0.001) (105). Moreover, a prospective cohort study confirmed the high predictive value for MALAT1 in differentiating patients with sepsis (106), indicating the potential of MALAT1 to be developed a biomarker to facilitating management in septic patients. In another study, Gui et al. concluded that lncRNA Antisense non-coding RNA in the INK4 locus (ANRIL)/miR-125a axis was upregulated in sepsis patients (107), which suggest that ANRIL could work as another potential biomarker for sepsis risk and severity. Several studies have suggested that lncRNA maternally expressed gene 3 (MEG3) might serve as a valuable indicator for the progression of sepsis, providing new strategy against acute respiratory distress syndrome in sepsis (108, 109).

Beyond that, several lncRNAs, such as tumor necrosis factor-related and heterogeneous nuclear ribonucleoprotein L-related immune-regulatory lncRNA (lnc-THRIL), intersectin 1-2, ZNFX1 antisense RNA, and highly upregulated in liver cancer (HULC), with strong correlations with risk, severity, and mortality of sepsis (110–113), were considered to play important roles in predicting prognosis in septic management. Though the results presented by the researchers were appealing, large-scale studies are lacking to ascertain real change in medical therapy for sepsis. Zheng et al. identified 14 lncRNA signatures for facilitating septic diagnosis using individualized pairwise analysis of gene expression (6). Nevertheless, the investigations to illuminate the specific mechanisms of these lncRNAs during sepsis are still lacking.

## LncRNAs in Sepsis-Induced Immune Cells Dysfunction

Since sepsis is regarded as a dysregulated host immune response to infection, which is mainly mediated by the inflammatory responses, it is hypothesized that certain lncRNAs play essential roles in regulating immune cells in sepsis pathogenesis. WAP four-disulfide core domain 21 (Wfdc21) was found to be specifically expressed in dendritic cells (DCs) (114). Though the research on Wfdc21 were few, one study has revealed the capacity of Wfdc21 to promote the differentiation of monocytes into DCs, as well as the T cell activation (114), providing supportive evidence related to Wfdc21 as a vital immune responses regulators. In LPS-stimulated macrophages, the expression level of Wfdc21 was elevated, and Wfdc21 was found to modulate the secretion of IL-1β and TNF-α through interacting with signal transducer and activator of transcription 3/TLR4 signaling pathway (90). Under different stimulations, macrophages were able to be polarized into M1 or M2 phenotype (115, 116). It has been illustrated that there is a close relationship between M1 phenotype and sepsis, while enhancing M2 polarization might produce anti-inflammatory cytokines and protect the body from sepsis progression (115). Knockdown of NEAT1 has been reported to promote macrophage M2 polarization by modulating miR125a-5p/TNF receptor-associated Factor 6 (TRAF6)/transforming growth factor-activated kinase 1 axis in septic inflammatory responses (77).

Plasmacytoma variant translocation 1 (PVT1) silencing was reported to ameliorate inflammation in macrophage during sepsis *via* downregulating p38/MAPK pathway (78). In particular, the inhibition of p38/MAPK signaling could also significantly reduce the elevation of PVT1 in sepsis (78). Taken together, the aforementioned studies indicated that lncRNA could exert therapeutic influence on sepsis through regulating the activities of macrophages. Beyond that, lncRNA such as MEG3 might serve as a promising target for septic diagnosis. In study conducted by Pan et al., the lower expression level of MEG3 in septic patients could predict sepsis occurrence with an AUC of 0.856 (75).

## LncRNAs in Sepsis-Induced Myocardial Dysfunction

Myocardial injury triggered by sepsis is characterized with impaired cardiac ejection function and myocardial contractility. However, the exact mechanism of this pathological process is not clear, and there is still a lack of effective therapeutic agents. It has been reported that lncRNAs could deteriorate sepsis development by damaging mitochondrial functions and activating inflammatory response. Mitochondrial functional impairments are known to seriously affect the energy metabolism of the body, leading to irreversible cardiomyocyte injury during sepsis (117). A recent study has revealed that SRY-box transcription factor 2 (SOX2) overlapping transcript (SOX2OT) was a crucial participant in exacerbating mitochondrial injury *via* the inhibition of SOX2 expression, which might gain new insight into septic cardiomyopathy therapy (83). Myocardial infarction associated transcript (MIAT) also exhibited the ability to promote mitochondrial dysfunction, by targeting miR-330-5p to regulate TRAF6/NF-κB signaling axis, which contributed to sepsis-induced cardiomyopathy (76). Interestingly, Ribonuclease mitochondrial RNA processing gene (RMRP) could decrease cardiomyocyte apoptosis and ameliorate mitochondrial damage, upregulating the expression of heat shock protein family A member 4 by competitive adsorption of miR-1-5p (79). Beyond inducing cardiomyopathy by damaging mitochondria, some lncRNAs took part in the progression of sepsis through interacting with NF-κB. HOTAIR was significantly upregulated in cardiomyocytes of septic mice, and positively correlated with the activation of NF-κB (35). In addition, NEAT1 knockdown could alleviate the myocardial injury induced by inflammatory response through the inhibition of TLR2-mediated NF-κB signaling (50). PVT1 knockdown in septic injured myocardial tissues promoted myocardial cell apoptosis by inhibiting Bcl-2 mediated signaling pathway (23), which exhibited its therapeutic potential as targets.

**Figure 2:**
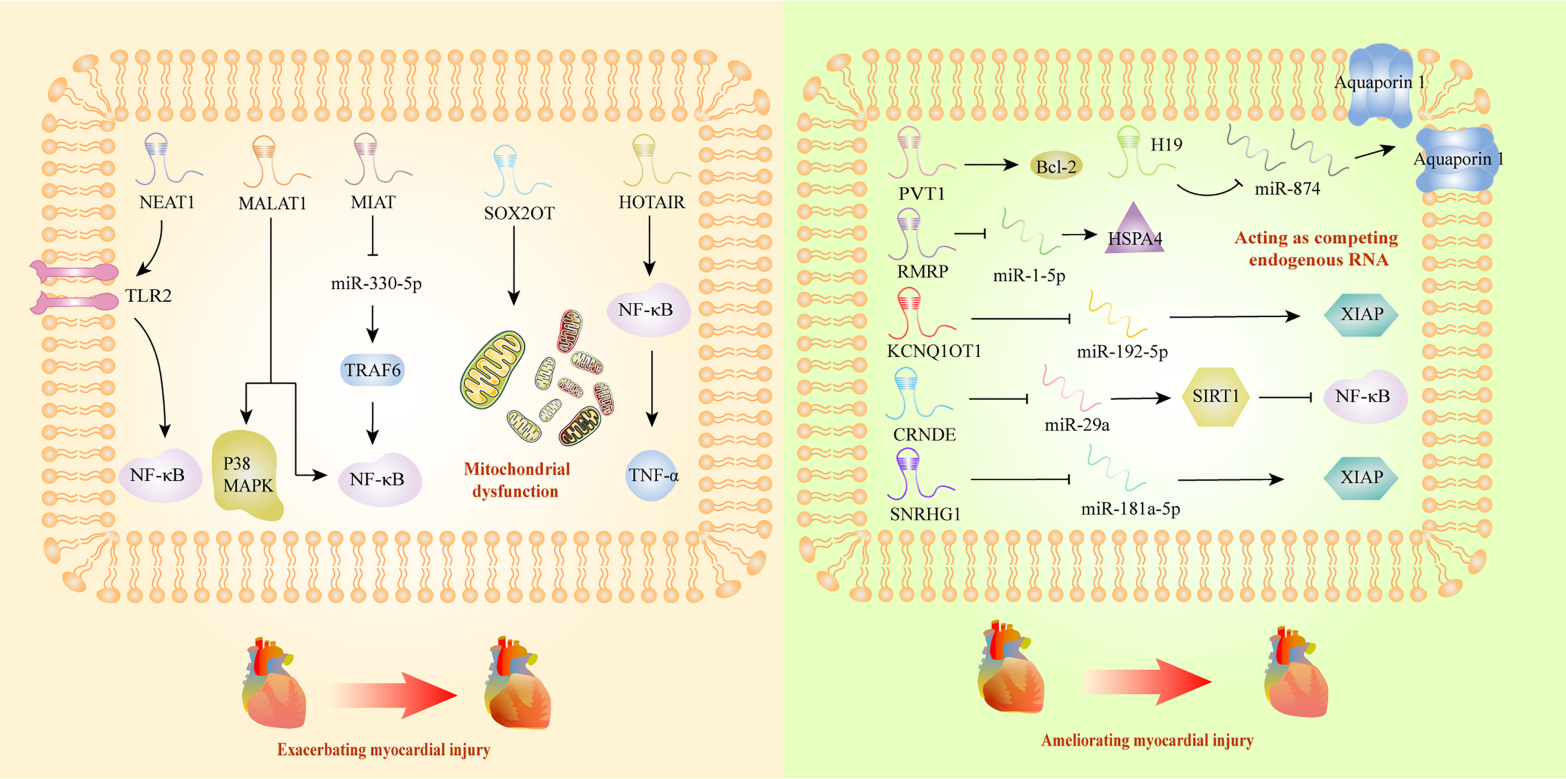
Long non-coding RNAs as potential biomarkers in sepsis-induced myocardial dysfunction. Bcl-2, B-cell lymphoma 2; CRNDE, colorectal neoplasia differentially expressed; HOTAIR, HOX transcript antisense RNA; HSPA4, HSP70 protein 4; KCNQ1, KCNQ1 overlapping transcript 1; MALAT1, metastasis-associated lung adenocarcinoma transcript 1; MAPK, mitogen-activated protein kinase; MIAT, myocardial infarction associated transcript; NEAT1, nuclear-enriched abundant transcript 1; NF-κB, nuclear factor κB; RMRP, RNA component of mitochondrial RNA processing endoribonuclease; SIRT1, sirtuin 1; SNRHG1, small nucleolar RNA host gene 1; SOX2OT, SOX2 overlapping transcript; TNF-α, tumor necrosis factor α; TLR2, Toll-like receptor 2; TRAF6, TNF Receptor-Associated Factor 6; XIAP, X-linked inhibitor of apoptosis protein.

Additionally, it has been reported that some lncRNAs affected sepsis-induced myocardial dysfunction in a protective way. LncRNA H19 could ameliorate myocardial injury induced by inflammatory response, and thus inhibited the expression of miR-874, promoting the expression of Aquaporin 1, and regulating the process of intracellular and extracellular water metabolism (67). In addition, researchers confirmed that KCNQ1 opposite strand/antisense transcript 1 might have cardioprotective impact on sepsis, through interacting with miR-192-5p/X-linked inhibitor of apoptosis protein (XIAP) axis (70). Moreover, CRNDE ameliorated the apoptosis in myocardial tissues by inhibiting miR-29a and promoting expression of sirtuin (SIRT) 1 (57). In vitro study, small nucleolar RNA host gene (SNHG) 1 was discovered to protect body against sepsis-induced myocardial injury through the modulation of miR-181a-5p/XIAP axis (81). In a word, lncRNAs might become new circulatory diagnostic markers and potential therapeutic targets for patients with septic-induced cardiac dysfunction (**Figure 2**).

## LncRNAs in Sepsis-Induced Lung Injury

**Figure 3:**
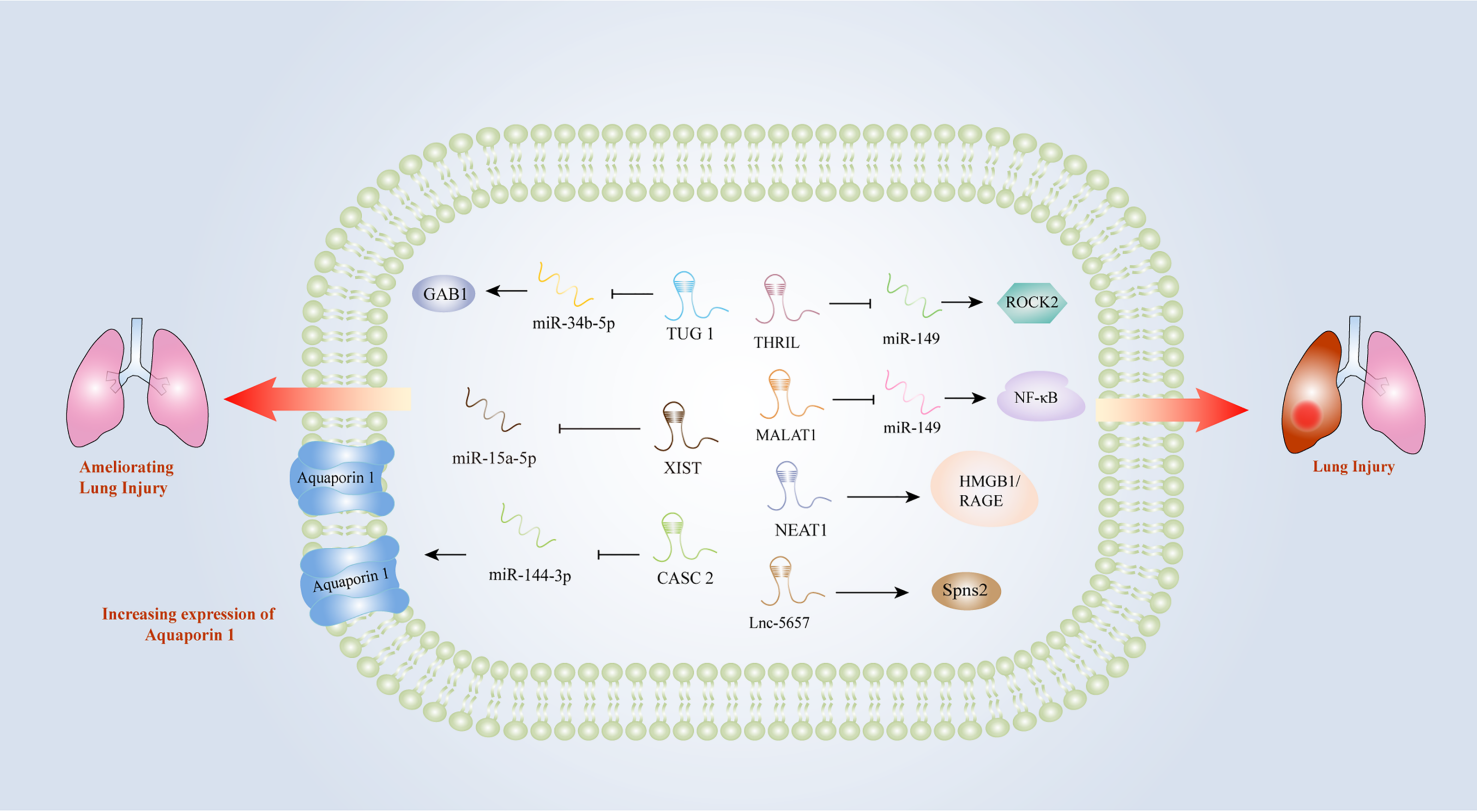
Long non-coding RNAs as potential biomarkers in sepsis-induced lung injury. CASC2, cancer susceptibility candidate 2; GAB1, GRB2 associated binding protein 1; HMGB1, high-mobility group box1; IL-1β, interleukin-1β; IL-6, interleukin-6; MALAT1, metastasis-associated lung adenocarcinoma transcript 1; MyD88, myeloid differentiation factor 88; NEAT1, nuclear-enriched abundant transcript 1; NF-κB, nuclear factor κB; RAGE, receptors for advanced glycation end products; ROCK2, Rho-associated coiled-coil containing protein kinase 2; THRIL, tumor necrosis factor and HNRNPL related immunoregulatory long non-coding RNA; TLR2, Toll-like receptor 2; TNF-α, tumor necrosis factor α; TUG1, Taurine up-regulated gene 1; XIST, X inactive-specific transcript.

Early intervention of acute respiratory distress induced by sepsis is essential for the treatment of patients with sepsis. However, there is still a lack of diagnostic markers for acute lung injury (ALI) induced by sepsis. An increasing number of studies have indicated that lncRNAs might be involved in the progression of acute respiratory distress triggered by sepsis. In the LPS-treated lung epithelial cell model, MALAT1 promoted inflammatory lung injury by activating myeloid differentiation factor 88 (MyD88)-mediated NF-κB signaling pathway through competitively binding to miR-149 (47). Knockdown of MALAT1 could significantly ameliorate the septic lung injury, by inhibiting p38 mitogen-activated protein kinase (MAPK)/p65 NF-κB signaling pathway (48). Investigators found that lnc-THRIL might accelerated lung injury through the inhibition of miR-424 and restoring Rho-associated kinase 2 (ROCK2) (88). Similarly, the investigation performed by Liu et al. identified a close relationship between lncRNA-5657 and sepsis-associated lung injury using high-throughput sequencing technology, demonstrating that the silence of lncRNA-5657 could protect lung from inflammation-induced tissue damage during sepsis (73). Contrary to the aforementioned studies, some lncRNAs exhibited protective capacities in lung injury evoked by sepsis. The upregulation of NEAT1 could inhibit NF-κB *via* activating HMGB1/RAGE pathway and reduced the inflammatory alveolar epithelial cell injury induced by LPS (52). In the mouse model of ALI induced by LPS, cancer susceptibility candidate (CASC) 2 was found to be overexpressed, which could reduce the apoptosis of lung epithelial cells by regulating aquaporin (63). TUG1 could also alleviate pulmonary inflammation and lung vascular endothelial cell injury (89). X inactive specific transcript (XIST) was observed to be overexpressed in a lung injury rat model, and the researchers concluded that XIST might ameliorate sepsis-induced lung injury by modulating miR-16-5p (91). Hence, lncRNAs play important roles in sepsis-induced lung injury and they may become new diagnostic and therapeutic biomarkers for lung injury caused by sepsis (**Figure 3**).

## LncRNAs in Sepsis-Induced Liver Injury

Hitherto, there have been very limited investigations with respect to the roles of lncRNAs in sepsis-associated liver injury, though the liver is regarded as a potential target of inflammatory response in sepsis. According to results of present studies, it is reasonable to speculate that some lncRNAs who work as important participants in liver tumors may also exert influences on sepsis-related liver injury. In a study that investigating the mechanism of NEAT1 in hepatocellular carcinoma tissues, NEAT1 could regulate the expression of epidermal growth factor receptor (EGFR), thereby contributing to cancer cell proliferation (118). In a sepsis-induced liver injury model NEAT1 was also observed to compete against Let-7a to release TLR4, promoting subsequent inflammatory response (36). Additionally, LINC00472, which was reported to act as a tumor suppressor in liver cancer (119), alleviated sepsis-triggered acute hepatic injury (AHI) *via* targeting the miR-373-3p/tripartite motif (TRIM) 8 axis. CRNDE was reported to promote tumorgenesis of hepatocellular carcinoma by the sponge of miR-203 (120), while Li et al. demonstrated that CRNDE played protective roles in AHI induced sepsis by regulating miR126-5p and BCL2L2 (58). Nevertheless, the research on the mechanisms of lncRNAs in sepsis-induced liver injury is still lacking, and more clues are needed for the treatment of sepsis.

## LncRNAs in Sepsis-Associated Encephalopathy

There is little research with regard to the mechanisms of lncRNAs in sepsis-associated encephalopathy (SAE). Sun et al. firstly identified lncRNAs in LPS-induced SAE rodent brains with RNA-seq, suggesting the potential roles that lncRNA acted as in inflammatory conditions of SAE (121). NEAT1 is considered to work as a pivotal role in sepsis-related organ dysfunction (36, 50, 119). Liu et al. demonstrated that the inhibition of NEAT1 could ameliorate sepsis-associated brain injury, which might work as a target for treating sepsis-triggered brain injury in future (54). LncRNA Lethe was revealed to protect against sepsis-induced brain injury *via* regulating autophagy in murine cortical neurons (71). Yin et al. provided *in vivo* evidence that the knockdown of SOX2OT alleviated neurogenesis impairment triggered by sepsis *via* inhibiting SOX2 level (84). Contrary to the study on LncRNA-5657 in sepsis-induced lung injury (73), the results of LncRNA-5657 in septic encephalopathy indicated that this lncRNA could reduce inflammatory response in SAE. However, due to the fact that these studies only provided preliminary evidence that lncRNAs might become potential targets for therapy and markers for diagnosis of SAE, further investigations are still warranted (122).

## LncRNAs in Sepsis-Induced Renal Dysfunction

Acute kidney injury (AKI) is one of the most common complications of sepsis, and approximately 50% to 60% of patients with sepsis have AKI. According to a recent meta-analysis, MALAT1, CASC2, TapSAKI, XIST, and HOXA cluster antisense RNA 2 (HOXA-AS2) were considered to be the potential predictive biomarkers and therapeutic targets of AKI (123). While the research that exploring the mechanisms of MALAT1 in sepsis-related renal dysfunction was lacking, CASC2 was observed to inhibit inflammation mediated by NF-κB signaling pathway in human renal tubular epithelial cells (65). In line with the previous study, the overexpression of CASC2 was discovered to protect against human or embryonic kidney cells damage induced by LPS *via* the regulation of miR-545-3p/PPARα axis (64). In vivo, overexpressed TapSAKI could facilitate inflammatory reaction and cell apoptosis in sepsis-associated kidney injury (85). This finding is in line with that of Zhang et al. who revealed that the knockdown of TapSAKI could ameliorate kidney injury, as a sponge of miR-205, that providing us a new promising target for treating sepsis (86). Being the downstream effector of miR-15a-5p, lncRNA XIST was upregulated in LPS-stimulated mouse model, which promoted cell apoptosis thereafter. Nonetheless, the exact role of XIST in sepsis-triggered kidney damage needed further investigations (92). Another key lncRNA, HOXA-AS2, was found to attenuate the progression of sepsis-induced renal dysfunction by inhibiting the activation of NF-κB signaling pathway (68).

Meanwhile, an increasing number of studies have shown that lncRNAs, such as HOTAIR and TUG1, have protective effects in the regulation of sepsis-induced AKI. For example, HOTAIR promoted the expression of Bcl-2 and inhibited the apoptosis of renal epithelial cells by negatively regulating miR-34a (37). TUG1 was discovered to play protective roles in the progression of sepsis-associated AKI, while a recent research showed silencing TUG1 could attenuate inflammation and apoptosis in renal ischemia-reperfusion injury model (124). However, some lncRNAs, such as NEAT1, play opposite roles in septic AKI. NEAT1 promoted NF-κB-mediated inflammatory responses by inhibiting the expression of miR-204 (53). Additionally, lncRNA TCONS_00016233 was observed to aggravate septic AKI *via* the modulation of the miR-22-3p/apoptosis-inducing factor mitochondrion-associated 1 axis, which indicated that TCONS_00016233 not only acted as a diagnostic marker but also as a new target for sepsis-induced AKI therapy (87). Similarly, lncRNA SNHG14 was overexpressed in septic patient plasma with AKI, and SNHG14 could interact with miR-495-3p and exert influence on cell apoptosis, proliferation, and inflammatory response, thereby exacerbates sepsis-induced AKI (82). RMRP was also discovered to become a contributor to AKI triggered by sepsis, providing a novel strategy for AKI therapy (80). Interestingly, CRNDE could sponge miR-181a-5p, eventually promoting the expression of NF-kB, and aggravated the kidney injury induced by sepsis (32), while downregulated LncRNA CRNDE was reported to increase miR-181a-5p and reduce PPARα by Wang et al., thereby aggravating inflammation in sepsis (61).

**Figure 4:**
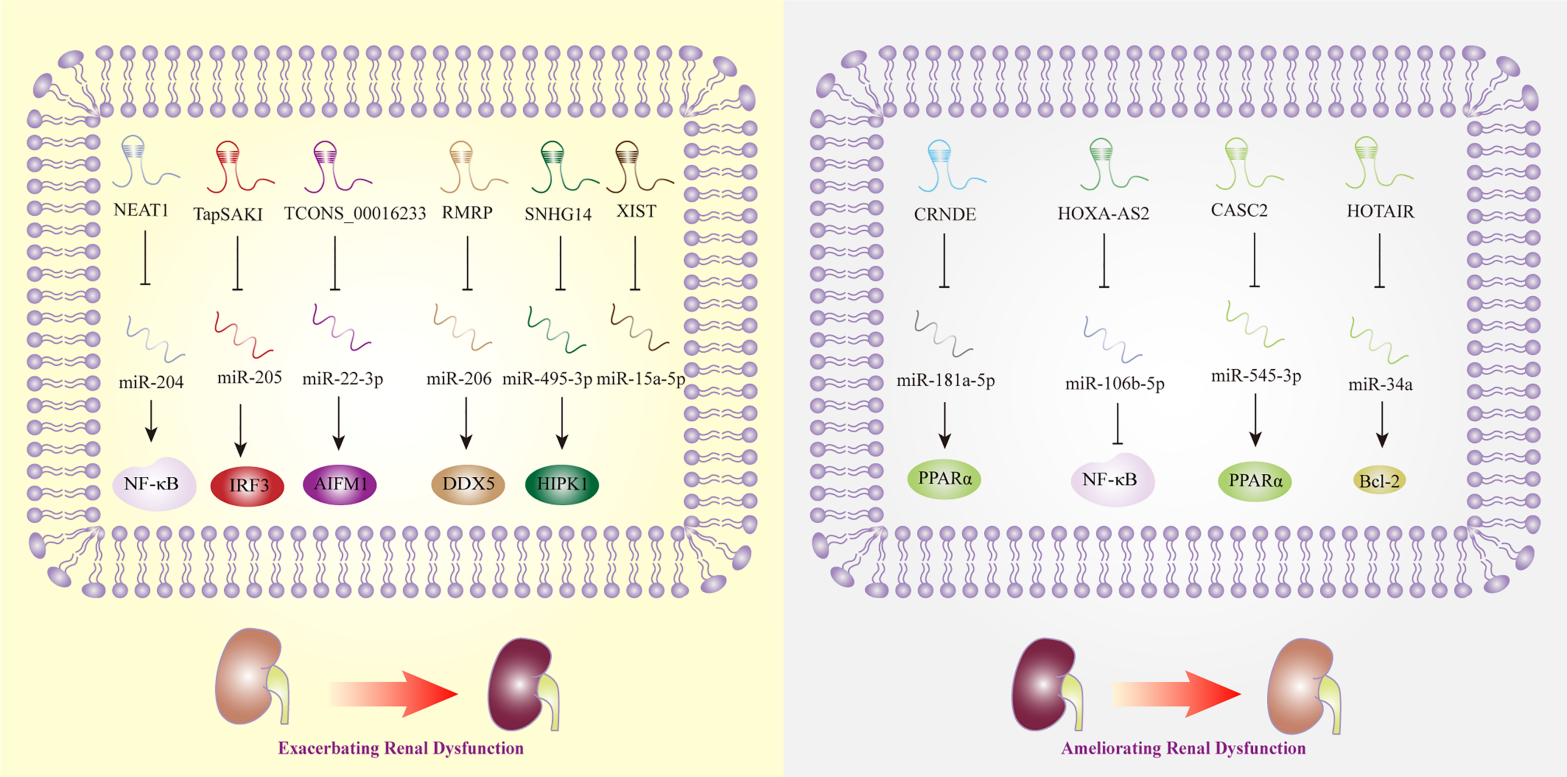
Long non-coding RNAs as potential biomarkers in sepsis-induced renal dysfunction. AIFM1, apoptosis-inducing factor mitochondrial -associated 1; Bcl-2, B-cell lymphoma 2; CASC2, cancer susceptibility candidate 2; CRNDE, colorectal neoplasia differentially expressed; DDX5, DEAD box polypeptide 5; HIPK1, homeodomain-interacting protein kinase 1; HOTAIR, HOX transcript antisense RNA; HOXA-AS2, HOXA cluster antisense RNA 2; IRF3, interferon regulatory factor 3; NEAT1, nuclear-enriched abundant transcript 1; NF-κB, nuclear factor κB; PPARα, Peroxisome proliferator-activated receptor-α; SNHG14, small nucleolar RNA host gene 14; TapSAKI, transcript predicting survival in AKI; XIST, X inactive-specific transcript.

Noted that the RNAs sequencing results extracted from human proximal renal tubular epithelial cells have revealed that the expression level of lncRNA linc-ATP13A4-8z was elevated significantly in plasma of patients with sepsis, which indicated the potential correlations of lncRNA linc-ATP13A4-8z with the progression of renal epithelial injury (125). Nevertheless, more work needs to be done to explore kidney-specific lncRNAs and to evaluate their credibility and effectiveness as biomarkers of sepsis in the future (**Figure 4**).

## LncRNAs in Sepsis-Induced Endothelial Cell Dysfunction

**Figure 5:**
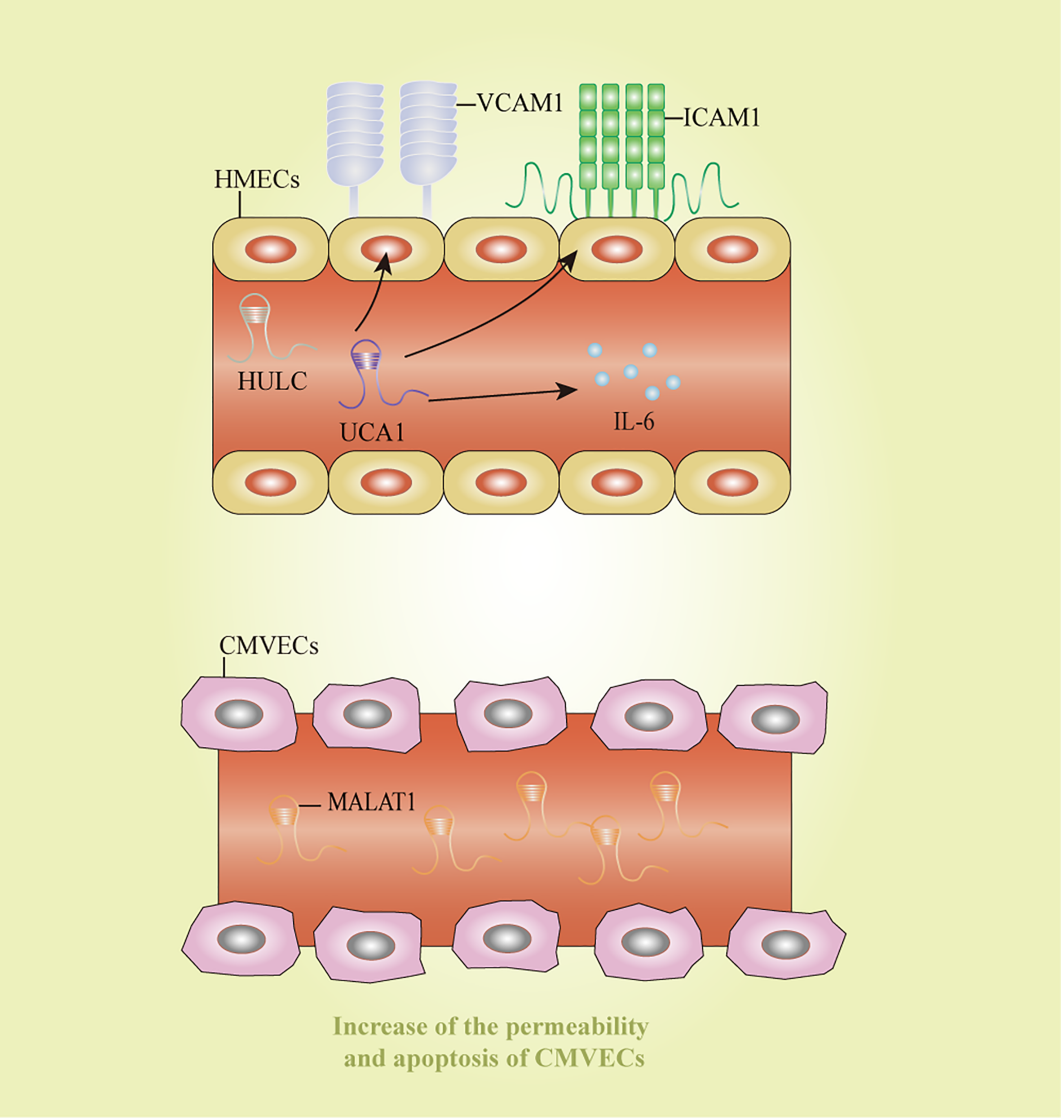
Long non-coding RNAs as potential biomarkers in sepsis-induced endothelial cell dysfunction. CMVECs, cardiac microvascular endothelial cells; HMECs, human mammary epithelial cells; HULC: highly upregulated in liver cancer; ICAM1: intracellular adhesion molecule 1; IL-6, interleukin-6; MALAT1, metastasis-associated lung adenocarcinoma transcript 1; UCA1, urothelial carcinoma-associated 1; VCAM1, vascular cell adhesion molecule 1.

Endothelial cell inflammation is a critical process in the pathogenesis of sepsis. HULC and urothelial carcinoma-associated 1 increased the expression of IL6, vascular cell adhesion molecule 1, and intracellular adhesion molecule 1 after LPS-induced inflammation in an endothelial cell model, thereby impairing vascular endothelial functions (126). In consistent with the previous study, HULC silencing could reverse the sepsis process stimulated by LPS by modulating miR-128-3p/Rac family small GTPase 1 axis in human umbilical vein endothelial cells (HUVECs) (69). Additionally, MALAT1 was significantly elevated in LPS-stimulated CMVECs, accompanied by the increase of permeability and apoptosis of CMVECs (46). TUG1 exerted protective effects on cell autophagy, apoptosis, and inflammatory reaction in HUVECs treated by LPS *via* inhibiting miR-27a-3p and then mediating the function of SLIT2 (55). These lncRNAs might shed lights on novel ideas for therapeutic strategies of endothelial cell dysfunction induced by sepsis. However, the clear mechanisms about how these lncRNAs affect endothelial cell functions remain unclear, and further research is still needed to discover more lncRNAs and clarify their regulatory mechanisms. Potential diagnostic and therapeutic markers for sepsis-induced endothelial cell dysfunction were displayed in **Figure 5**.

## LncRNAs in Gastrointestinal System During Sepsis

Most current studies addressed the values of lncRNAs as predictor markers, diagnostic markers, and therapeutic targets for gastrointestinal cancers (127). Hitherto, few studies have investigated the roles of lncRNAs in gastrointestinal system during sepsis. In a recent research, the expression of lncRNA Gas5 was found to be decreased in mice that developed gut bacteria-associated sepsis after being exposed to γ-rays. It was reported that the reduced lncRNA Gas5 expression and the elevation of miR-222 expression might be associated with the polarization of macrophages in mesenteric lymph nodes influenced by the irradiation (66). The administration of glycyrrhizin might protect mice with gastrointestinal acute radiation syndrome against subsequent sepsis through the modulation of macrophage polarization and the inhibition of miR-222 expression (66).

Sepsis is a life-threatening disease with the potential to be develop into severe systemic inflammation, and the gut is one of the major target organs. The gastrointestinal tract is one of the most critical organs damaged in sepsis. Increasing evidence revealed that the disruption of microbiome could predispose to sepsis and exert negative influences on sepsis outcomes (30, 128). On the other hand, sepsis is closely related to disruption of the intestinal barrier (129), and improving intestinal barrier function might be a new focus for further investigations and clinical treatment. Intestinal tract is another important target organs in the development of sepsis, with the capacity of inducing multiple organ injuries eventually. Nonetheless, the investigations on the mechanisms of lncRNAs in gastrointestinal dysfunction induced by sepsis is relatively limited. Hence, it could be conceivably hypothesized that lncRNAs that play critical roles in intestinal inflammatory disorder such as inflammatory bowel disease, might also have important effects on sepsis-induced gastrointestinal dysfunctions. Improving the function of intestinal barrier function might also provide a novel focus on for future investigators and clinical treatment. Hence, certain lncRNA with the capacity of modulating gastrointestinal inflammation and repairing intestinal barrier might contribute to the development of sepsis.

B cell-activating factor (BAFF) was elevated in sepsis patients (62). Blocking BAFF was confirmed to play protective effects on intestinal barrier function *via* modulating NF-κB/MLCK/MLC signaling pathway in endotoxemia mouse (62). CDKN2B-AS1 was found to enhance the function of intestinal barrier (130). In an LPS-induced intestinal inflammation model, seven core lncRNAs and their target genes were identified and analyzed using a co-expression network, which provided new insights into the pathological mechanism for intestinal inflammation (131). In another inflammatory cell model, NEAT1 was observed to promote the translocation of NF-κB p65, mediating intestinal inflammation by the regulation of TNF superfamily member 1B (132). Knockdown of SNHG5, that could interact with miR-375/Janus kinase 2 axis, reduced the apoptosis and promoted the proliferation of mouse colon cells (133). In another ulcerative colitis murine model, TUG1 suppressed intestinal epithelial cell apoptosis, thereby inhibited the progression of ulcerative colitis (134).

## The Challenge of lncRNAs as Biomarkers and Therapeutic Targets

LncRNAs are involved in a variety of signaling pathways with diverse and complex biological functions. Recent years, a large number of literatures have indicated that lncRNAs played critical roles in regulating the process of sepsis, which were expected to become promising diagnostic biomarkers and therapeutic targets in the future. However, the clinical values for these lncRNAs have not been fully confirmed, and thus their transformation into the clinical process may encounter many challenges.

Although techniques such as Real-Time PCR, second-generation sequencing, and microarray analysis have been used to detect the expression levels of lncRNAs and their targeting signaling pathway in sepsis, most of these measurements or detections were merely at the experimental stages. Thus the research to explore the feasibility of lncRNAs as biomarkers in clinical practice is still lacking. Currently, numerous investigations revealed the effects of lncRNAs on modulating the progression of sepsis both *in vivo* and vitro. Nevertheless, considering the differences between humans and animals, there are still much difficulties existing in the transformation of experimental outcomes into clinical practice. It is of great significance to explore the roles of lncRNAs participated in the pathological mechanism of sepsis. Although many investigations have suggested the possible roles for lncRNAs in sepsis, the exact mechanisms for lncRNAs in the development of sepsis are still unclear. Hence, further research is needed to verify the specificity and feasibility for lncRNAs as novel specific biomarkers and therapeutic targets in sepsis patients, ultimately providing the new insight into targeted therapy of sepsis.

## Conclusions

In conclusion, available evidence revealed that lncRNAs were strongly associated with pathophysiological process of sepsis. Additionally, lncRNAs were specifically expressed and exerted different influences in sepsis-induced organ dysfunctions, which presented the potential of providing basis for targeted therapy. However, the precise mechanism of how lncRNAs affect the development of sepsis has remained unclear, and the current results of relevant studies are still inconsistent. Future research should focus on elucidating the molecular mechanisms of lncRNAs and actively exploring organ-specific lncRNAs as therapeutic targets and diagnostic markers for organ dysfunction in sepsis.

## Author Contributions

CW, JH, and XL contributed to conception and design of the study. CW, GL, JS, HK, and DW wrote the first draft of the manuscript. CW, GL, JS, and HK drew the figures. XL finalized and supervised the whole project. All authors contributed to the article and approved the submitted version.

## Funding

This work was supported by National Natural Science Foundation of China (81972204), Natural Science Foundation of Guangdong Province (2019A1515011097), Innovation Program of Shenzhen (Grant No. JCYJ20180508165208399), Science and Technology Planning Project of Guangzhou (201904010089), the grant from the State Key Lab of Respiratory Disease, Guangzhou Medical University (SKLRD-Z-202002), and the 111 Project (D18010) from the Ministry of Education of China.

## Conflict of Interest

The authors declare that the research was conducted in the absence of any commercial or financial relationships that could be construed as a potential conflict of interest.

## Publisher’s Note

All claims expressed in this article are solely those of the authors and do not necessarily represent those of their affiliated organizations, or those of the publisher, the editors and the reviewers. Any product that may be evaluated in this article, or claim that may be made by its manufacturer, is not guaranteed or endorsed by the publisher.
